# Health in the Schoolroom

**Published:** 1895-10-26

**Authors:** 


### Social Problems.

### HEALTH IN THE SCHOOLROOM.

Health is made or marred in youth, in the nursery and the schoolroom. The marvellous recuperative power of youth, which seems to overcome the effects of limitless tarts and of any wetting gained in the service of the goddess of sport, should not blind us to the fact that, according to the care or neglect shown in his upbringing, a strong child's health will be weakened or a weak one's improved. In his earliest months and years the child of well-to-do parents has all the advantage. Good clothes, a well-warmed and ventilated nursery, regular exercise, food restricted to his digestive powers both in kind and in amount, set him off on his school career in the best possible condition. In the home he has all the advantages, but what about the school?

The national school is generally a comparatively new building, designed by an architect who has specially studied the requirements of such a building. It is under Government inspection, and any faults in ventilation or sanitation, any overcrowding, any lack of conveniences must be remedied at once, under penalty of withdrawal of more or less of the grant on which the maintenance of the school depends. If the homes of the poor were as good as their schools they would scarcely have an excuse for an epidemic. Of course these schools are intended solely for education. The children eat their meals and sleep at home, and play in the streets after school hours, so that they have many and varied opportunities for injuring their health; but, as far as the schoolroom itself is concerned, we are sure that the most expensive institution in the kingdom is no better than a good ordinary national school. Indeed, the sites of our old public schools were chosen so frequently for any rather than hygienic reasons, that it is much to the credit of their directors if they are on this point satisfactory at all.

But the school to which the sons of the middle and upper classes are sent are more than mere educational institutions; for at least nine months in the year they are the children's homes. Not only the schoolroom but the boarding-house, the dormitory, the diet-sheet, must be considered, and beyond these the moral influences which surround the pupil. It is one of the boldest things we do, we English, to send our sons at their most impressionable age away from the refining and purifying influences of home life. So strong are the arguments against it that, as Mr. R. H. Quick—himself a master in a public school—declares, nothing could justify it except its success. It succeeds in its main object, in making men of the boys—such trifles as the power to make Latin verses, some knowledge of Greek, a certain unwilling acquaintance with Euclid, and even a little English being thrown in. But are they good men or not?—that is a point not to be ignored.

It is impossible to bring together a number of boys without some of them being bad; all one can ask is that the influence of the bad boys shall be less than that of the good. In some schools there goes down a tradition of evil, in others a tradition of good. The influence of the head and other masters is great in this matter, but in spite of the best efforts it is difficult to keep a boys' school from being the haunt of vile and unnatural sins. Supervision will do much to prevent these evils, the constant inculcation of a true ideal of a manhood will do more, but even then the aid of the architect must not be despised. A great deal depends on the internal arrangements of the dormitory. Open dormitories are in general use for young boys, and are in every way the best for all. Boys, taking them as a whole, are not vicious, and the evil inclinations of one are restrained by the public opinion of all, where publicity is possible. But in many schools the elder boys are privileged to have "studies" which are also their bed-rooms. Sometimes these are complete rooms, sometimes only cubicles; but in either case they are bad. The room which is used both as bed-room and sitting-room is inevitably ill-ventilated, and the occupant inclines to go to bed straight from work, to broken and unrefreshing slumber. In the cubicle the evil is only varied. There is indeed a certain circulation of air above the partitions, but of air that has been breathed and rebreathed, and is valueless for health. In the cubicle, moreover, an idle boy has exceptional power to annoy a studious neighbour without the knowledge of the authorities; and, which is worse, it affords only a limited obstacle to the association of boys for evil purposes, while it affords them all the secrecy they desire. For every reason, then, the cubicle is the worst form of sleeping-room, while the open dormitory of moderate size, under the supervision of a teacher or monitor, is the best. The study should be a day-room only, in order that it and the dormitory may be properly ventilated. Trying to combine the two injures the value of both.

Concerning food, there are two things to be kept in view—it should be plain and it should be ample. Of course boys may injure themselves by over-eating, but it is not plain beef and mutton, bread and milk, that tempt them to excess. And growing boys, with the strain of study and exercise to meet, require plenty of food. We have heard of a public school of good repute where the meals were so scanty as to leave the boys hungrily dependent on home supplies. This is unjust, for the pupils' parents pay enough to provide for a sufficiency of food for them, and it is injurious to the boys, whether it keeps them fasting or sends them to spend all their pocket-money at the confectioner's. Beer is still given in some schools. This hardly seems necessary. Most boys can digest their food without its aid, and anything of the nature of a stimulant had better be given only under doctor's orders.

There is one point on which a great deal must be left to the teacher's discretion, although in case of doubt an early appeal to the doctor is wise. We mean the decision as to whether apparent stupidity on the part of any pupil is due to mere inattention or to some defect of mind or flaw in sight and hearing. The heavy-handed discipline of old days is now largely a thing of the past, but cases now and again arise of a boy being punished for deficiencies for which he is in no way responsible. Where doubt exists it is always wise to call in the doctor. Then myopia and deafness will be put on the right footing; incorrigible dulness, whether due to clearly-marked brain disease or not, will find its proper place; and the schoolmaster can exercise a more unhesitating control over those that remain.

The question of exercise may in general be left to the boys themselves. To some boys games are as detestable as the *pons asinorum* is to others, and the qualities of each must be left to modify his neighbour's. That is what boys are sent to school for—to learn by example the *juste milieu* of things; to develop their own powers indeed, but to pick up also so much of the characteristics of others as will enable them to hold their own amid the "all sorts" which go to make a world, and to look without too much complacency on the virtues which they possess or too much contempt on the lack of them in their fellows.

